# Defense Enzyme Responses in Dormant Wild Oat and Wheat Caryopses Challenged with a Seed Decay Pathogen

**DOI:** 10.3389/fpls.2017.02259

**Published:** 2018-01-23

**Authors:** E. Patrick Fuerst, Matthew S. James, Anne T. Pollard, Patricia A. Okubara

**Affiliations:** ^1^Department of Crop and Soil Sciences and Western Wheat Quality Laboratory, Washington State University, Pullman, WA, United States; ^2^Department of Crop and Soil Sciences, Washington State University, Pullman, WA, United States; ^3^USDA-ARS Wheat Health, Genetics and Quality Research Unit, Washington State University, Pullman, WA, United States

**Keywords:** *Avena fatua*, polyphenol oxidase, exochitinase, seed decay, soil seed-bank, ecophysiology, weed biocontrol, *Fusarium avenaceum*

## Abstract

Seeds have well-established passive physical and chemical defense mechanisms that protect their food reserves from decay-inducing organisms and herbivores. However, there are few studies evaluating potential biochemical defenses of dormant seeds against pathogens. Caryopsis decay by the pathogenic *Fusarium avenaceum* strain *F.a*.1 was relatively rapid in wild oat (*Avena fatua* L.) isoline “M73,” with >50% decay after 8 days with almost no decay in wheat (*Triticum aestivum* L.) var. RL4137. Thus, this fungal strain has potential for selective decay of wild oat relative to wheat. To study defense enzyme activities, wild oat and wheat caryopses were incubated with *F.a*.1 for 2–3 days. Whole caryopses were incubated in assay reagents to measure extrinsic defense enzyme activities. Polyphenol oxidase, exochitinase, and peroxidase were induced in whole caryopses, but oxalate oxidase was reduced, in response to *F.a*.1 in both species. To evaluate whether defense enzyme activities were released from the caryopsis surface, caryopses were washed with buffer and enzyme activity was measured in the leachate. Significant activities of polyphenol oxidase, exochitinase, and peroxidase, but not oxalate oxidase, were leached from caryopses. Defense enzyme responses were qualitatively similar in the wild oat and wheat genotypes evaluated. Although the absolute enzyme activities were generally greater in whole caryopses than in leachates, the relative degree of induction of polyphenol oxidase, exochitinase, and peroxidase by *F.a*.1 was greater in caryopsis leachates, indicating that a disproportionate quantity of the induced activity was released into the environment from the caryopsis surface, consistent with their assumed role in defense. It is unlikely that the specific defense enzymes studied here play a key role in the differential susceptibility to decay by *F.a*.1 in these two genotypes since defense enzyme activities were greater in the more susceptible wild oat, compared to wheat. Results are consistent with the hypotheses that (1) dormant seeds are capable of mounting complex responses to pathogens, (2) a diversity of defense enzymes are involved in responses in multiple plant species, and (3) it is possible to identify fungi capable of selective decay of weed seeds without damaging crop seeds, a concept that may be applicable to weed management in the field. While earlier work on seed defenses demonstrated the presence of passive defenses, this work shows that dormant seeds are also quite responsive and capable of activating and releasing defense enzymes in response to a pathogen.

## Introduction

Weeds cause global crop losses through reduced yield, nutrient, and water competition, and by harboring damaging pests and pathogens (Oerke, 2006). Financial losses due to yield decline can be catastrophic, with weeds causing an estimated $20 billion annually in crop damage losses in the U.S. (Pimentel et al., 2005). Despite much effort and research aimed at developing cultural and chemical weed control methods, weeds continue to reduce crop yields. One reason for the persistence of weeds in agricultural systems is the longevity of viable seeds in the soil, known as the soil seed-bank. The number of weed seeds per square meter of agricultural soils often exceeds 10,000 (Baskin and Baskin, 2006) with many of these seeds remaining viable for years or decades (Gallagher and Fuerst, 2006; Baskin and Baskin, 2014; Long et al., 2015). Wild oat (*Avena fatua* L.) is one of the 10 worst annual weeds of temperate regions due to its persistence in the soil seed-bank, abundant seed production, and multiple generations (times of emergence) during the growing season (Beckie et al., 2012). Use of herbicides against wild oat has resulted in many cases of herbicide resistance (Owen and Powles, 2009).

In an increasingly eco-conscious global community, it is important to consider biological methods for controlling weeds. Seed decay has been defined as “A process in which the physical integrity of a seed is degraded, ultimately leading to death” (Long et al., 2015). The role of microorganisms to manage the weed seed-bank by enhancing decay has been approached theoretically (Kremer, 1993; Chee-Sanford et al., 2006). Metagenomics and other molecular and statistical methods under development may improve our understanding of the microbial characteristics of weed-suppressive soils (Müller-Stöver et al., 2016). In the soil, microbial activity plays a part in plant nutrient uptake (Gyaneshwar et al., 2002), signaling (Harrison, 2005; Babikova et al., 2013), and resistance to biotic and abiotic stress (Yang et al., 2009; Pozo et al., 2010). Certain microbial community members have beneficial roles in root health through plant growth promotion or biocontrol of pathogenic microbes (Yin et al., 2013). *Trichoderma* spp. are root-colonizing and plant growth-promoting fungi that are widely studied for their ability to suppress diseases such as those caused by *Fusarium oxysporum* in onion (*Allium cepa* L.) and tomato (*Solanum lycopersicum* Mill.) (Abdelrahman et al., 2016; Jogaiah et al., 2017). However, many other microorganisms are considered pathogenic and detrimental to seed viability, germination, and seedling growth (Bressan, 2003). Rhizobacteria have been shown to selectively suppress downy brome (*Bromus tectorum* L.) seedling growth in wheat (Kennedy et al., 2001). Pathogenic fungi can be lethal to seeds of the weed, downy brome (Meyer et al., 2008), and cause significant decay in wild oat caryopses *in vitro* by the seed-pathogenic fungus *Fusarium avenaceum F.a*.1, the subject of this study (De Luna et al., 2011; Fuerst et al., 2011).

Seeds have been shown to contain constitutively-expressed antimicrobial compounds called phytoanticipins (Dalling et al., 2011) and wheat (*Triticum aestivum* L.) caryopses have been shown to contain defense enzymes and proteins in the extrinsic tissue layers of the caryopsis (Jerkovic et al., 2010) that may help the seed resist pathogen attack in the soil. Indeed, the tenacious glumes and floral bracts of wild emmer wheat have been shown to store and release defense enzymes upon hydration that may protect the caryopsis (Raviv et al., 2017). Prior research on wild oat indicated that *F.a*.1 induced the release of the defense enzyme polyphenol oxidase (PPO) into the medium surrounding the seed, suggesting the dormant caryopses had active sensing and response mechanisms to pathogen attack (Anderson et al., 2010; Fuerst et al., 2011, 2014). Proteomics observations suggested that chitinase (CHI) and oxalate oxidase (OxO) proteins also were released into the medium surrounding the wild oat caryopsis (Anderson et al., 2010).

Based on these observations we hypothesized that several plant defense enzyme activities in addition to PPO would be induced and released in response to the *F.a*.1 pathogen. Among the numerous plant pathogenic response proteins known (Pandey et al., 2016), CHI and OxO were chosen based on the preliminary observations mentioned (Anderson et al., 2010), whereas peroxidase (POD) was selected based upon its widely studied role in plant defense responses and in the pathogen-induced oxidative burst (Lamb and Dixon, 1997).

The potential roles of these enzymes in seed defense have been previously discussed (Jerkovic et al., 2010; Fuerst et al., 2014). All four enzymes participate in cell wall-associated host defense (Hücklehoven, 2007), the first line of defense against pathogen invasion, and all are expressed in seeds. PPO uses molecular oxygen to produce *o*-quinones and melanins and may inhibit pathogens and predators by creating lignin-like physical barriers and possibly toxic products (Yoruk and Marshall, 2003). PPO accumulates in host tissue in response to wounding (War et al., 2012). Class III PODs are secreted plant proteins with a remarkable number of functions including cross-linking cell wall polymers and lignification (Almagro et al., 2009; Cosio and Dunand, 2009). Their role in defense is due to strengthening cell walls and massive production of reactive oxygen species. OxOs have dual defense activities including the catabolism of fungal-derived oxalic acid, a metabolite toxic to plants, and production of fungicidal levels of H_2_O_2_ (Lane, 2002). OxO was induced by powdery mildew in barley and confers host resistance in barley against the white mold pathogen *Sclerotinia* (Dumas et al., 1993, 1995). Chitinases hydrolyze polymers containing N-acetylglucosamine such as chitin found in fungal cell walls, and have been associated with antifungal activity (Yan et al., 2008; Grover, 2012). Chitinases were among the earliest pathogenesis-related (defense) proteins to be identified (Legrand et al., 1987; Seidl, 2008).

In previous work with wild oat, we showed that most of the PPO activity induced by *F.a*.1 was released into the leachate, whereas in untreated wild oat most of the activity was retained on the caryopsis surface (Fuerst et al., 2011). However, the relative contributions of the caryopsis and fungal pathogen to PPO activity were not quantitated, which we evaluated here. We hypothesized that PPO, CHI, POD, and OxO would be induced and released (solubilized) in response to *F.a*.1 in both wild oat and wheat. We also hypothesized that enzymatic activity responses would be similar qualitatively, if not quantitatively, in these particular wild oat and wheat genotypes and that activities of these enzymes would be correlated with resistance to decay. Wheat was chosen because it is the major cereal crop infested with wild oat in Pacific Northwest agroecosystems; the relative decay response of wild oat and wheat caryopses to *F.a*.1 is critical for understanding the potential for selective decay of weed and not crop seeds in the field. The selection of just one genotype of each species precludes any generalizations about any differences observed between the two species.

## Materials and methods

### Incubation of fungal cultures and caryopses

The fungal strain used in this study was *F. avenaceum* isolate *F.a*.1 (Fuerst et al., 2011), originally referred to as isolate 223a (De Luna et al., 2011). *F.a*.1 cultures were started by placing either an agar plug of actively growing mycelia or a piece of cellulose filter disc containing dried fungal mycelia inoculum directly on the center of a petri dish containing potato dextrose agar (PDA) (Okubara et al., 2013). *F.a*.1 cultures were incubated at 25°C for 1.5–2 weeks, until cultures were ~6 cm in diameter. Colonies were visually screened for absence of contamination and sectoring.

Wild oat caryopses from the extremely dormant isoline Montana 73 (M73) (Naylor and Fedec, 1978) and caryopses from the moderately dormant red wheat cultivar RL4137 (Noll et al., 1982), raised under greenhouse conditions, were used in this study. Wild oat hulls were removed by hand. All caryopses were washed three times for 10 min in sterile water containing 0.02% Tween-20 by incubating for 10 min on an end-over-end shaker (Roto-Shake Genie Model SI-1100; Scientific Industries, Bohemia, NY). Wild oat or wheat caryopses (20–50 per dish) were placed at or near the leading edge of the growing fungal cultures. An equivalent number of caryopses were also placed on PDA as a control treatment. Unless otherwise indicated, fungal cultures with wild oat caryopses were incubated for 3 days at 15°C and plates with wheat caryopses were incubated at 23°C for 2–3 days. The higher temperature for wheat was required to prevent germination. Caryopses showing any sign of decay or germination were not used for enzyme assays. Caryopses were then subjected to whole-kernel enzyme assays, further processed for use in leachate assays, or evaluated for decay. A separate study demonstrated that enzyme activities and responses were generally similar following incubation at the two temperatures in wheat and wild oat (data not shown), and therefore qualitative comparisons of enzyme activities in the two species are acceptable.

### Caryopsis decay

**Figure 1:**
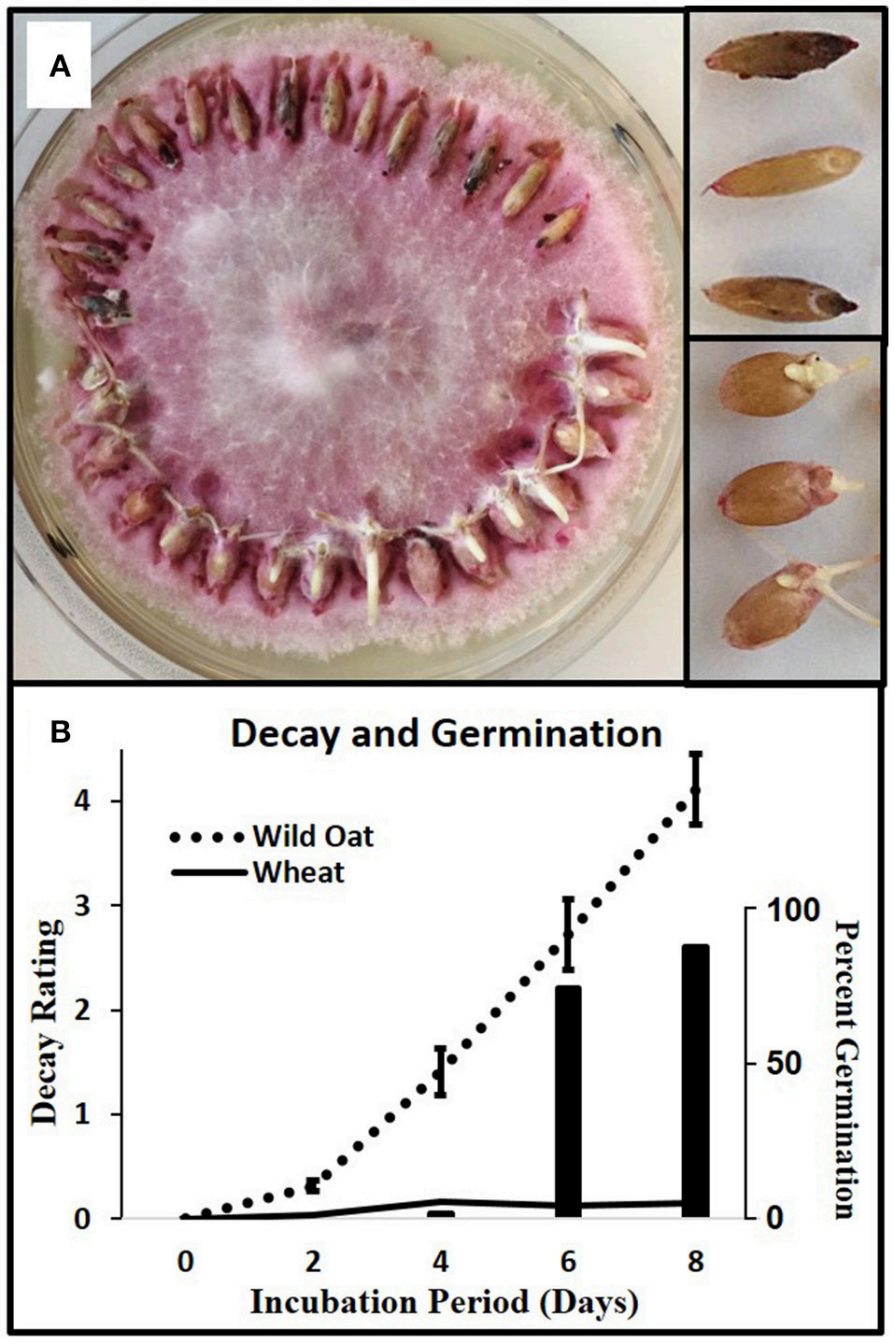
Caryopsis decay in wild oat and wheat. **(A)** Wild oat and wheat incubating on *F.a*.1, day 8, with mycelia removed from the tops of caryopses to improve visualization; close-up of decaying wild oat (upper right) and germinating wheat (lower right). Decay appears as darkened spots or areas. **(B)** Solid and dotted lines are decay ratings (left axis) for wild oat and wheat, respectively; wheat germination plotted as histogram (right axis). The decay rating ranged from zero (no decay) to 7 (completely decayed) as described in Methods and Materials. Error bars are the standard error of the mean.

For each of four replicates, 15 wild oat caryopses and 15 wheat caryopses were placed around the perimeter of *F.a*.1 cultures (Figure 1). Cultures were incubated at 25°C and decay ratings were taken at 2, 4, 6, and 8 days. The decay rating scale was adapted from De Luna et al. (2011): 0 = no decay, 1 = caryopsis tip turning dark, 2 = caryopsis showing a few dark spots and/or whole embryo dark, 3 = caryopsis showing multiple and/or large dark lesions, 4 = decay advancing to rest of caryopsis, more than just spots, 5 = 50% of caryopsis dark, 6 = 75% of caryopsis dark, and 7 = 100% of caryopsis dark.

### Generation of leachates

Caryopsis leachates were prepared from wild oat and wheat. Three replicates of 50 caryopses per treatment (PDA control vs. *F.a*.1) were placed in tared 15-mL centrifuge tubes to obtain caryopsis weight. To each tube, 7.5 mL 50 mM MOPS-T (3-morpholinopropane-1-sulfonic acid, containing 0.02%, v/v, Tween-20) pH 6.5 was added. Suspensions were incubated for 20 min at room temperature on an end-over-end shaker. Leachates were passed through a glass fiber filter (Whatman GF/A, 1.6 μM pore size; Whatman, Inc., Piscataway, NJ) and a polyethersulfone filter (0.45 μm pore size; Olympus Plastics, Genesee Scientific, San Diego, CA) to remove particulates and stored at −80°C.

Mycelial leachates were prepared from *F.a*.1 incubated alone, with wild oat caryopses, or with wheat caryopses, referred to as *F.a*.1-untreated mycelia, *F.a*.1-wild oat mycelia, and *F.a*.1-wheat mycelia, respectively. Fungal mycelia were collected from under the caryopses of *F.a*.1-wild oat and *F.a*.1-wheat treatments. The outer 2-cm of mycelia was scraped using a scalpel and transferred to a 15 mL centrifuge tube. Three petri dish replicates of each type of mycelia were leached for 20 min as previously described for caryopsis leachates, above, before centrifuging at 2,000 × *g* for 10 min. Leachate supernatants were decanted through glass wool and then passed through two additional filters as described above, before storing at −80°C.

### Defense enzyme assays

#### Whole caryopsis assays

General Procedure: Whole caryopsis enzyme activities were assayed spectrophotometrically based on the “whole kernel” method for PPO in wheat (Anderson and Morris, 2001; AACC International, 2010, Approved Method 22-85) with modifications. There were four petri dish replicates except where otherwise indicated. Caryopses were gently removed from PDA or the fungal mycelial bed with forceps. Five caryopses per replicate plate were transferred to a tared 2-mL microcentrifuge tube and samples were re-weighed. For each assay, a no-substrate control (five caryopses in buffer only) was run to determine background absorbance from leached caryopses; an additional control was substrate with no caryopses. All experiments were repeated and the results of the final experiment are presented. Results are reported as nmol gfwt^−1^ (grams fresh weight of caryopses) min^−1^.

For PPO assays, caryopsis samples were incubated in 1.25 mL substrate solution consisting of 10 mM L-DOPA (L-3,4-dihydroxyphenylalanine) in 50 mM MOPS-T. Samples were mixed and incubated at room temperature on an end-over-end shaker (Labquake model 415110, Barnstead/Thermolyne) for 25 min and 300 μL tropolone (2-hydroxy-2,4,6-cycloheptatrien-1-one; final concentration 1 mM) was added to terminate the reaction (Fuerst et al., 2006). Samples were then centrifuged at 10,000 × *g* for 1.5 min to remove particulate contaminants and 300 μL of supernatant was transferred to a microtiter plate. Absorbance at 475 nm was determined with a spectrophotometer (BioTek Epoch; BioTek Instruments, Inc.; Winooski, VT) and moles product were determined using an extinction coefficient of 3600 M^−1^ cm^−1^.

The CHI assay (exochitinase, β-N-acetylglucosaminidase) was based on the Sigma Chitinase Assay Kit (Catalog Number CS0980) in which the substrate, 4-nitrophenyl N-acetyl-β-D-glucosaminide, is hydrolyzed to release *p-*nitrophenol, which turns yellow at high pH. Caryopsis samples were incubated in 1 mL substrate solution consisting of 1 mg/mL substrate in 50 mM citrate buffer pH 4.8 with 0.02% (v/v) Tween-20. Samples were incubated as described above for 60 min. After centrifugation, 100 μL of supernatant was transferred to wells of a microtiter plate containing 200 μL sodium carbonate (final concentration 260 mM; final pH 10.7) kill solution. Absorbance at 405 nm was determined and moles product were determined using an extinction coefficient of 18000 M^−1^ cm^−1^.

Initial POD assays revealed a high degree of variability in activity in both wild oat and wheat caryopses. Therefore, we utilized eight replicates to increase statistical power. POD activity was assayed with a commercial assay kit (KPL ABTS 2-Component Peroxidase Substrate System, SeraCare Life Sciences, Milford, MA) in which the ABTS [2,2′-azino-bis(3-ethylbenzothiazoline-6-sulphonic acid)] concentration was 55 μM and the H_2_O_2_ concentration was 3 mM in a proprietary buffer with the addition of 0.02% (v/v) Tween-20. Caryopsis samples were incubated in 1 mL ABTS-H_2_O_2_ substrate solution and the no-substrate control was incubated in 50 mM citrate pH 4.1. Samples were incubated as described above for 30 min, after which 500 μL of 20% SDS (sodium dodecyl sulfate) was added to kill the reaction. After centrifugation, 300 μL of supernatant was transferred to a microtiter plate, and bubbles were eliminated with a light spray of ethanol. Absorbance at 418 nm was determined (Porstmann et al., 1981; Holm, 1995) and moles product were determined using an extinction coefficient of 36,000 M^−1^ cm^−1^.

Whole caryopsis OxO assays were based on the procedure of Requena and Bornemann (1999) which measures OxO-catalyzed H_2_O_2_ release by coupling the reaction to horseradish peroxidase-mediated ABTS oxidation. We report these activities as “apparent OxO activity” because the assay is indirect, and other factors affecting H_2_O_2_ concentration will affect the measured activity. The substrate solution consisted of 50 mM citrate pH 4.0, 20 mM oxalic acid pH 4.0, 1.8 mM ABTS, 0.02 mg/mL horseradish peroxidase (2.4 U/mL; Sigma # P-8125), and 0.02% Tween-20. Caryopsis samples were incubated in 1 mL substrate solution for 4 or 10 min (wild oat or wheat, respectively). Reactions were terminated by adding 0.5 mL 3% SDS in 30 mM citrate pH 2.8. Reaction tubes were centrifuged and 300 μL of supernatant was transferred to a microtiter plate. Absorbance at 418 nm was determined and moles product were determined using an extinction coefficient of 36,000 M^−1^ cm^−1^.

#### Leachate assays

Substrate buffers, kill solutions, and absorbance determinations were the same as described for whole caryopsis assays. Reactions were conducted in microtiter plates containing 150 μL (PPO) or 100 μL (CHI, POD, and OxO) of leachate. For CHI, POD, and OxO assays, 50 μL of a pH correction buffer was added to each well since leachates were made in pH 6.5 MOPS-T buffer, and the CHI, POD, and OxO assays were conducted at pH 4.8, 4.1, and 4.0, respectively. The correction buffer was 50 mM citrate pH 4.3 for CHI assay and 50 mM citrate pH 3.5 for POD and OxO assays. Reactions were initiated by adding 150 μL of substrate solution, mixing with the pipettor; total reaction volume was 300 μL. The microtiter plate was covered and incubated with shaking (800 rpm “Multi Microplate Genie,” Scientific Industries, Inc., Bohemia, NY) for 60 min at room temperature and reactions were terminated by adding 50 μL of kill solution. Leachate results are reported on both a gfwt and mg protein basis.

##### Protein assays

Protein content was determined in duplicate 75 μL leachate samples with the method of Bradford (1976) utilizing the “Quick Start Bradford Protein Assay Kit” (Bio-Rad Laboratories, Los Angeles, CA).

### Statistical analysis

Statistical analyses were conducted using SAS version 9.4 (SAS Institute, Cary, NC). Model assumptions were checked, and ANOVA was conducted using PROC GLM, PROC MIXED, and PROC UNIVARIATE. Treatment means were compared using the least significant difference (LSD) test in PROC MIXED with the PDIFF option in the LSMEANS statement. LSMEANS were separated using the PDMIX 800 macro (Saxton, 1998) at α ≤ 0.05.

## Results

### Seed decay

Wild oat showed a steady progression of decay symptoms from 2 to 8 days whereas wheat showed almost no decay (Figure 1). Wheat started germinating after 4 days due to its low level of dormancy relative to wild oat; thus this pathogen isolate causes the desired selective decay of this weed isoline in this crop variety.

### Caryopsis enzyme activities and protein

**Figure 2:**
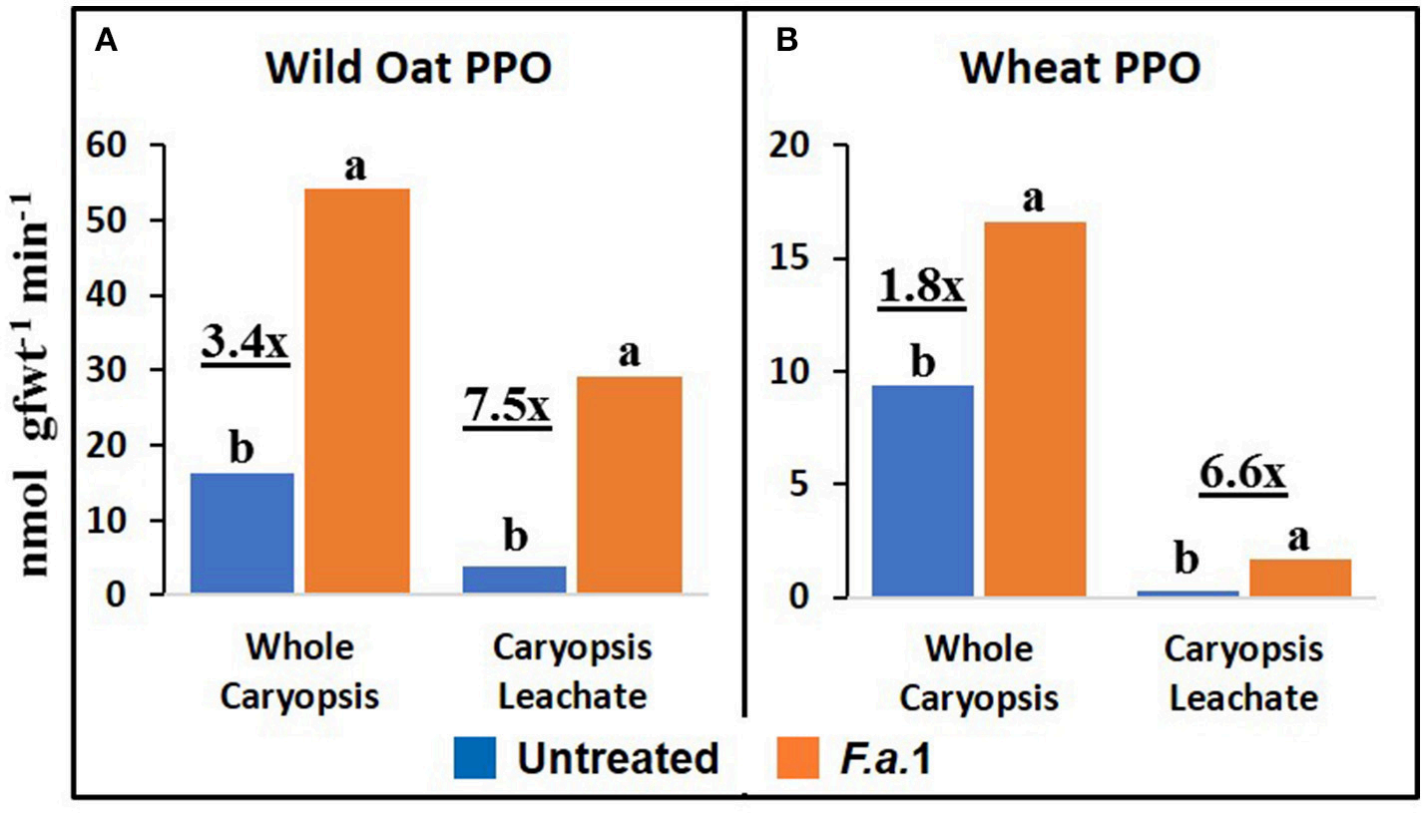
Polyphenol oxidase (PPO) activity in untreated and *F.a*.1-treated whole caryopsis and caryopsis leachate assays in wild oat **(A)** and wheat **(B)**. Units are nmol product per gram fresh weight of caryopses per minute. A pair of bars having different lower case letters are significantly different at *P* = 0.05 by the LSD test. Numbers followed by “x” represent the ratio of *F.a*.1-treated activity to untreated activity.

**Figure 3:**
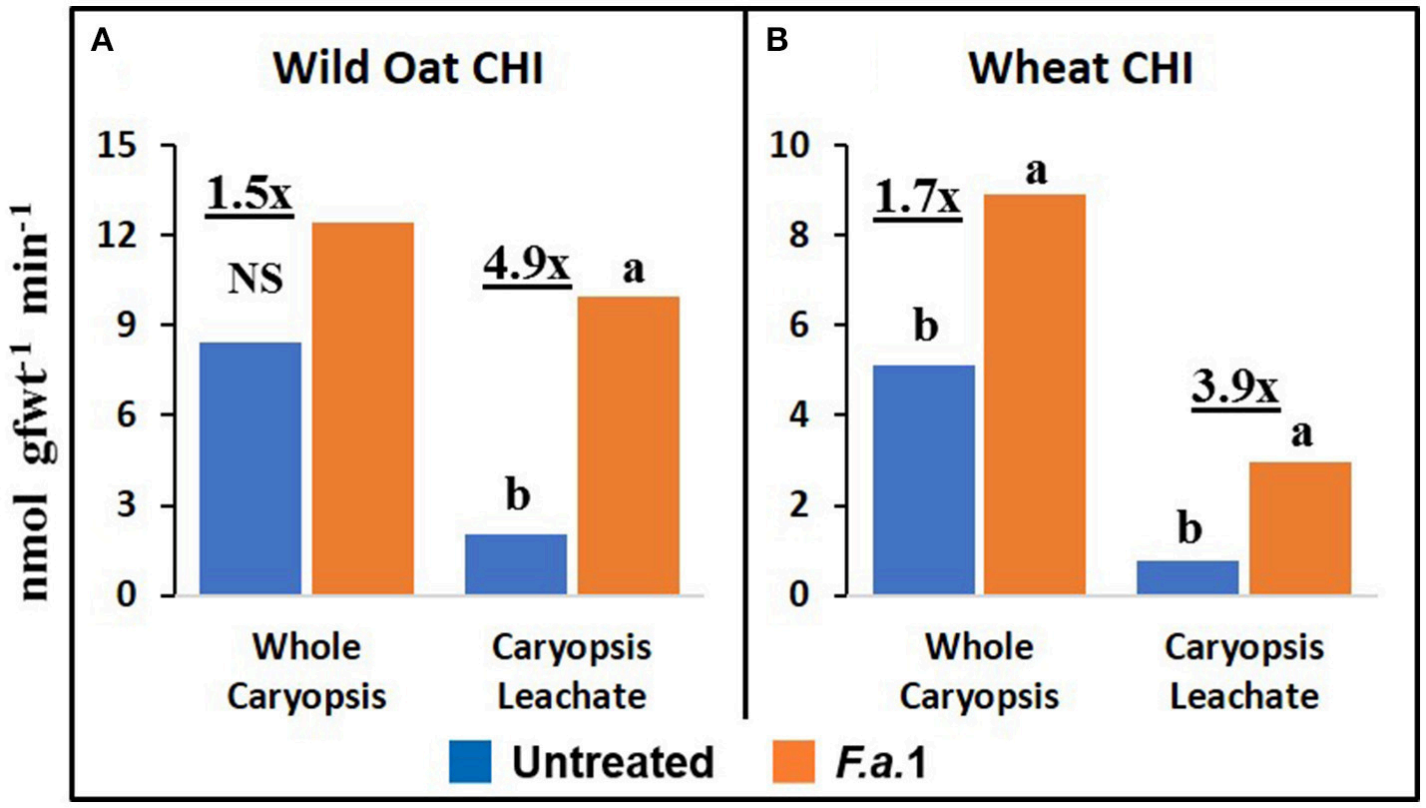
Exochitinase (CHI) activity in untreated and *F.a*.1-treated whole caryopsis and caryopsis leachate assays in wild oat **(A)** and wheat **(B)**. Units are nmol product per gram fresh weight of caryopses per minute. A pair of bars having different lower case letters are significantly different at *P* = 0.05 by the LSD test. NS, non-significant. Numbers followed by “x” represent the ratio of *F.a*.1-treated activity to untreated activity.

**Figure 4:**
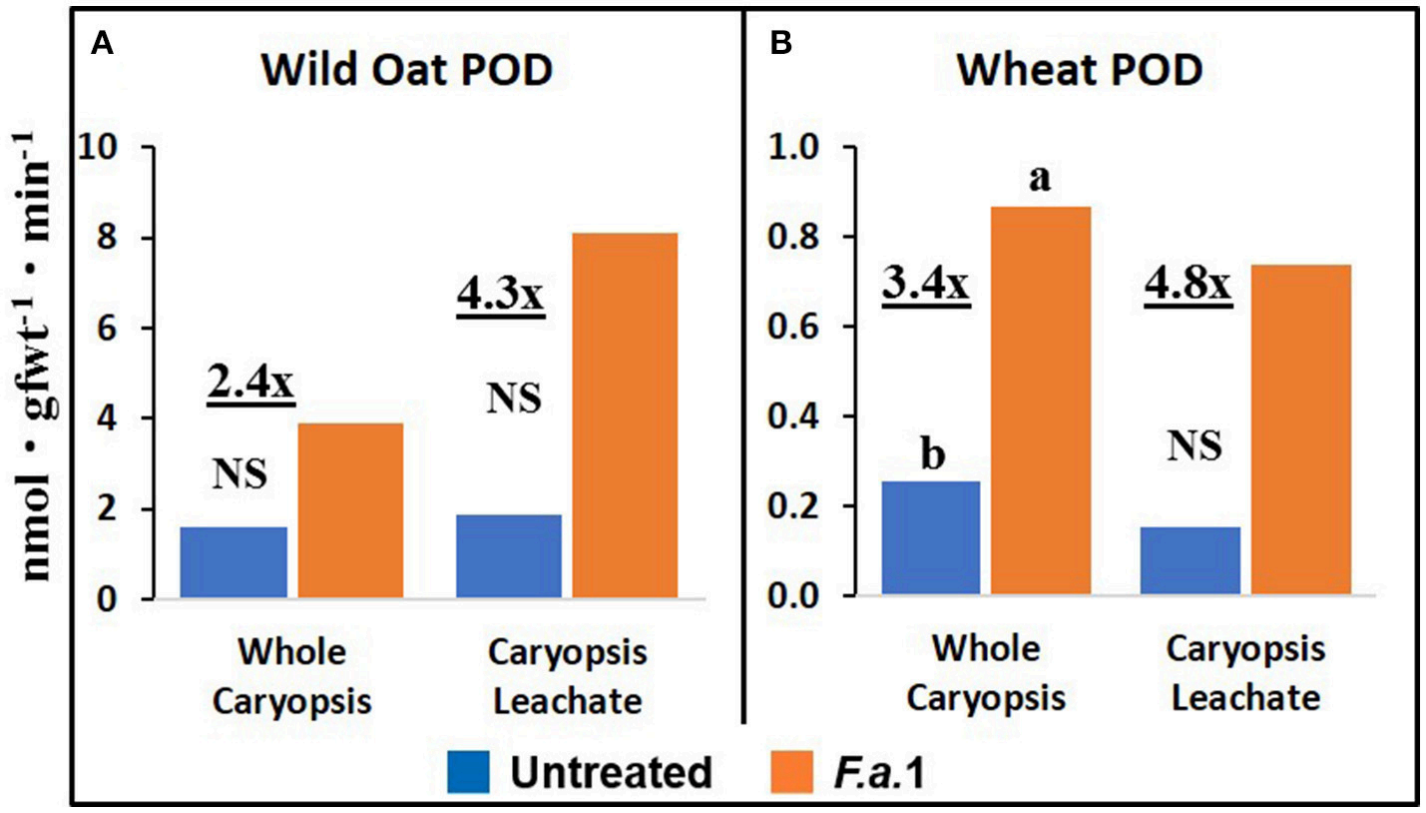
Peroxidase (POD) activity in untreated and *F.a*.1-treated whole caryopsis and caryopsis leachate assays in wild oat **(A)** and wheat **(B)**. Units are nmol product per gram fresh weight of caryopses per minute. A pair of bars having different lower case letters are significantly different at *P* = 0.05 by the LSD test. NS, non-significant. Numbers followed by “x” represent the ratio of *F.a*.1-treated activity to untreated activity.

**Figure 5:**
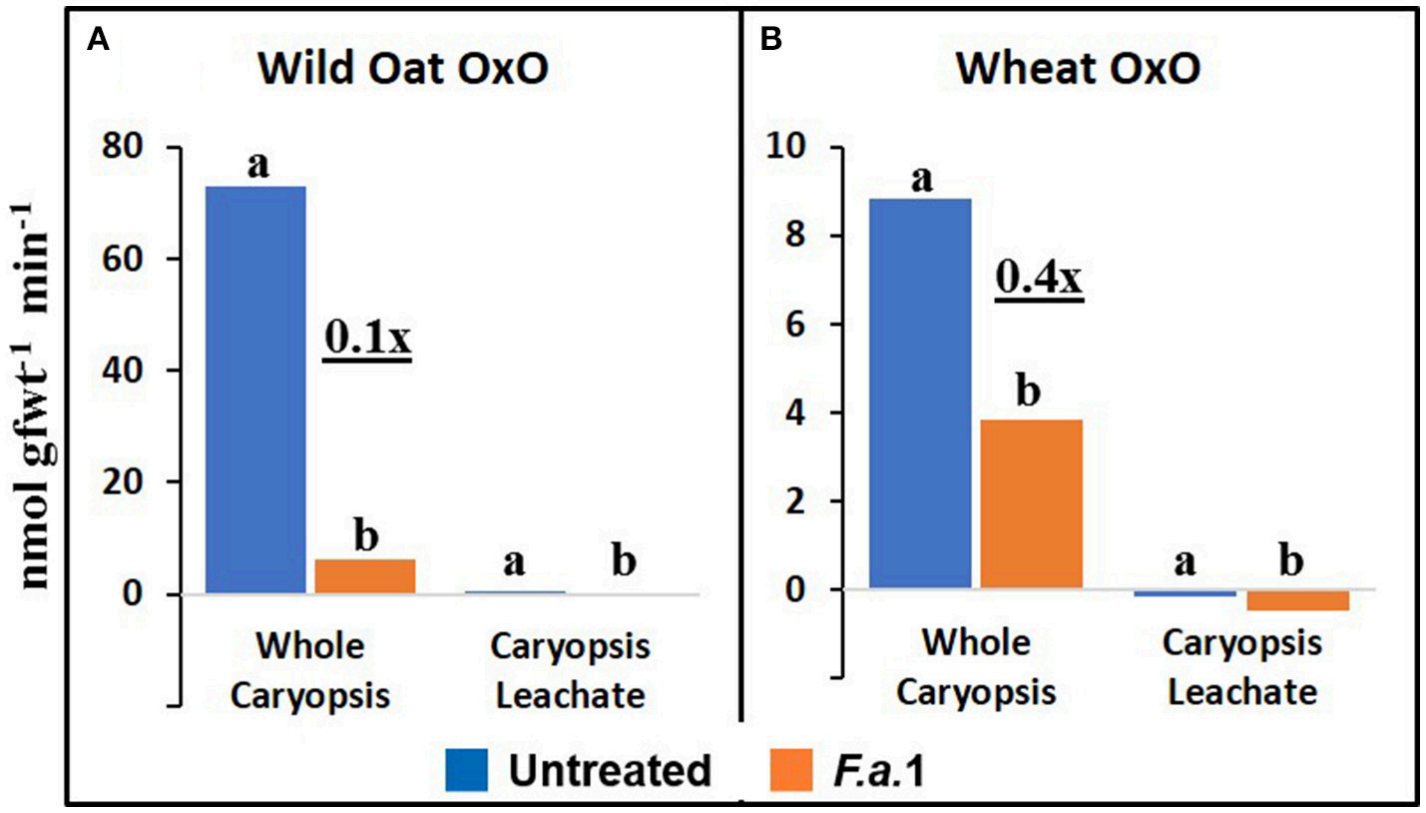
Apparent oxalate oxidase (OxO) activity in untreated and *F.a*.1-treated whole caryopsis and caryopsis leachate assays in wild oat **(A)** and wheat **(B)**. Units are nmol product per gram fresh weight of caryopses per minute. A pair of bars having different lower case letters are significantly different at *P* = 0.05 by the LSD test. Wheat leachate OxO activities were slightly negative after subtracting appropriate blanks **(B)**. Numbers followed by “x” represent the ratio of *F.a*.1-treated activity to untreated activity.

**Figure 6:**
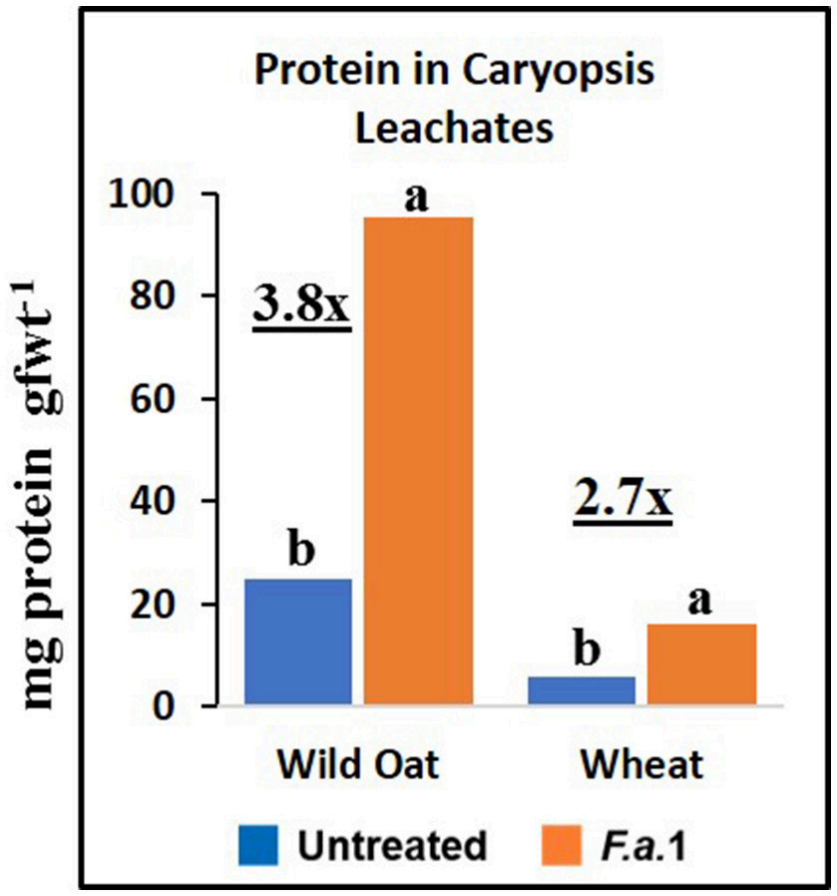
Protein content in untreated and *F.a*.1-treated caryopsis leachates. Units are mg protein per gram fresh weight of caryopses. A pair of bars having different lower case letters are significantly different at *P* = 0.05 by the LSD test. Numbers followed by “x” represent the ratio of *F.a*.1-treated activity to untreated activity.

The effect of *F.a*.1 on enzyme activities was qualitatively similar but quantitatively different between wild oat isoline M73 and wheat var. RL4137. *F.a*.1 exposure of caryopses, relative to untreated caryopses, induced PPO activity 3.4-fold in whole caryopses and 7.5-fold in leachates of wild oat (Figure 2A); the corresponding values for wheat were 1.8- and 6.6-fold, respectively (Figure 2B). Thus, the induction levels in leachates were greater than in whole caryopses. With *F.a*.1 challenge, CHI activity increased 1.5-fold in whole caryopses and 4.9-fold in leachates of wild oat (Figure 3A) compared to 1.7- and 3.9-fold, respectively for wheat (Figure 3B). Again, the level of CHI induction by *F.a*.1 was greater in leachates than in whole caryopsis assays. POD assays had extreme levels of variability, and consequently, there were few significant differences in response to *F.a*.1 (Figure 4). Nonetheless, the trend of greater induction levels in leachate than in whole caryopses was similar to that seen for PPO and CHI. *F.a*.1 exposure increased POD activity 2.4-fold in whole caryopses and 4.3-fold in leachates of wild oat (Figure 4A); the corresponding values for wheat were 3.4- and 4.8-fold, respectively (Figure 4B). Apparent OxO activity was reduced by *F.a*.1 treatment in whole caryopsis assays of wild oat and wheat (Figure 5), the opposite of the other defense enzymes. Apparent OxO activity in leachates was <1% of whole caryopsis activities (Figure 5); this indicates that nearly all the observed OxO activity was tightly bound to the caryopsis surface. Protein content in leachates increased 3.8-fold in wild oat and 2.7-fold in wheat following *F.a*.1 exposure of caryopses (Figure 6). This indicates that a significant amount of protein was solubilized following exposure to *F.a*.1.

PPO, CHI, POD, and apparent OxO activities were expressed constitutively in all whole caryopsis and caryopsis leachate assays except OxO leachate assays, as noted above (Figures 2–5). Enzyme activities were generally greater for this wild oat isoline than for this wheat variety for both whole caryopsis and leachate assays, which is indicated by the greater magnitude of the vertical scale for wild oat vs. wheat (part “A” vs. part “B,” respectively; Figures 2–5) for all enzymes. Since these data are expressed on caryopsis fresh weight basis, much of the difference between the species is the caryopsis weight. The average weight of a wheat caryopsis was 43 vs. 19 mg for wild oat, or 2.2-fold greater in wheat. Since the number of caryopses was the same in each assay, if results had been expressed on a per caryopsis basis instead of a weight basis, wheat values would have increased 2.2-fold relative to wild oat. With this correction for weight, wild oat values still exceeded those for wheat except that whole caryopsis CHI activity was somewhat greater in wheat than in wild oat (data not shown).

The magnitude of activities in whole caryopsis assays was generally greater than in caryopsis leachate assays (Figures 2–5). For the example of PPO in wild oat, untreated and *F.a*.1-treated whole caryopsis activities were 16.2 and 54.3 nmol gfwt^−1^ min^−1^, respectively, but were reduced to 3.9 and 29.2 nmol gfwt^−1^ min^−1^, respectively, in caryopsis leachates (Figure 2A). However, there was an exception to this generalization in the case of *F.a*.1-treated wild oat POD leachate activity (8.1 nmol gfwt^−1^ min^−1^) which was greater than whole caryopsis activity (3.9 nmol gfwt^−1^ min^−1^; Figure 4A).

### Defense enzyme activities in mycelial vs. caryopsis leachates

**Figure 7:**
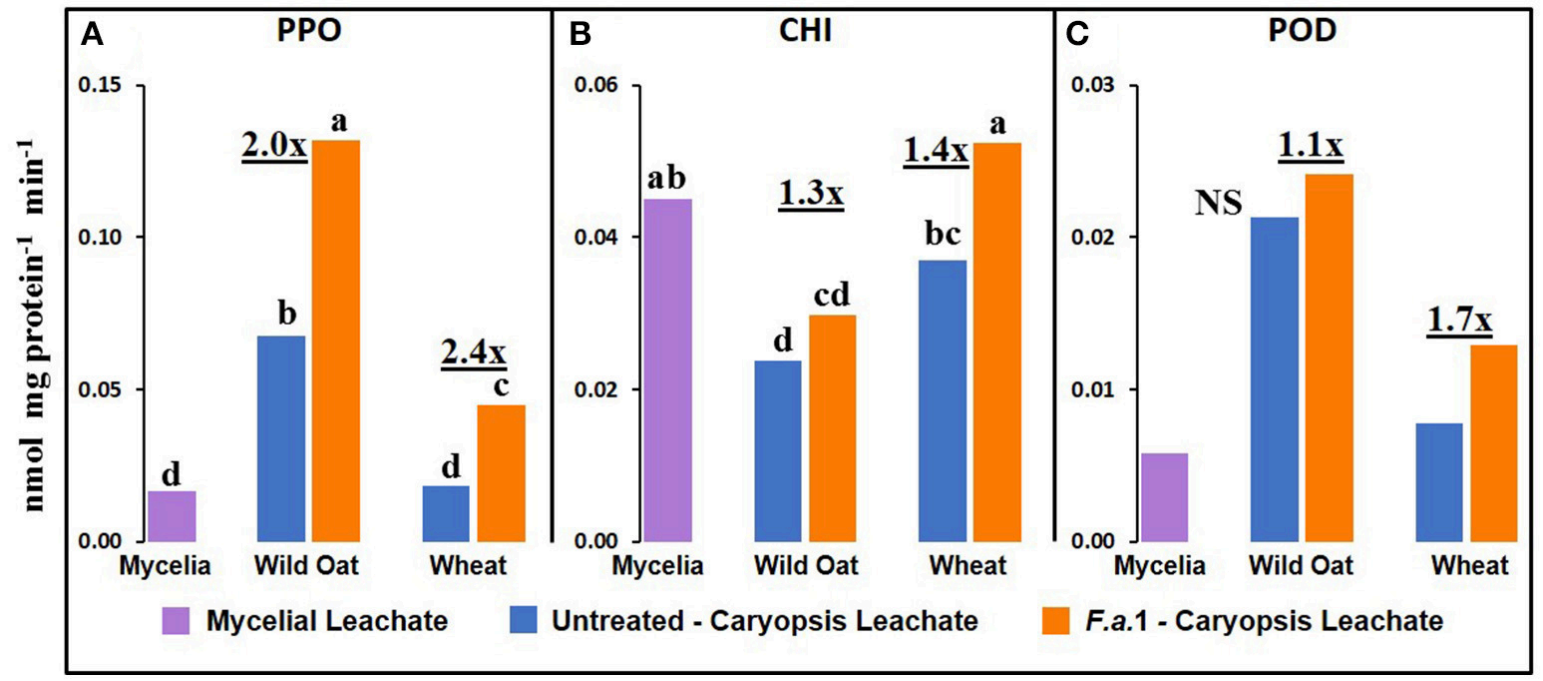
Defense enzyme activities on protein basis in *F.a*.1 mycelial leachate, and in untreated and *F.a*.1-treated caryopsis leachates **(A)** polyphenol oxidase, **(B)** exochitinase (CHI), and **(C)** peroxidase (POD). Units are nmol product per mg protein per minute. Bars not having the same lower case letter are significantly different at *P* = 0.05 by LSD; NS, non-significant. Numbers followed by “x” represent the ratio of *F.a*.1 activity to untreated activity in caryopses.

Although most mycelia were removed from caryopses before assays, the contribution of residual mycelia to the heretofore reported activities in *F.a*.1-treated caryopses (Figures 2–5) must be considered. It is difficult to discern the fungal vs. plant contributions to enzymatic activity but one potential indicator can be obtained by putting activities of both mycelial and caryopsis leachates on a protein basis. There was measurable PPO, CHI, and POD activity in *F.a*.1 mycelial leachates (Figure 7) while apparent OxO activity was negligible (data not shown). Therefore, it is probable that some portion of PPO, CHI, and POD activities observed in *F.a*.1-treated caryopses (Figures 2–4) can be attributed to fungal origin. The mycelial activity on a protein basis was lower than the caryopsis leachate activity for both PPO (Figure 7A) and POD (Figure 7C), suggesting that most of these activities in whole caryopsis and caryopsis leachate assays would probably have come from caryopses. However, in the case of CHI (Figure 7B), the activity in mycelial leachate was comparable to caryopsis leachates. Therefore, part of the whole caryopsis and caryopsis leachate CHI activities should be attributed to a mycelial source. However, a significant part of CHI activity in caryopsis assays must have also come from caryopses, considering that there was significant CHI activity in untreated caryopsis leachates of both wild oat and wheat (Figure 7B), i.e., in the absence of mycelia, and that the quantity of mycelia retained on caryopses was relatively small.

**Figure 8:**
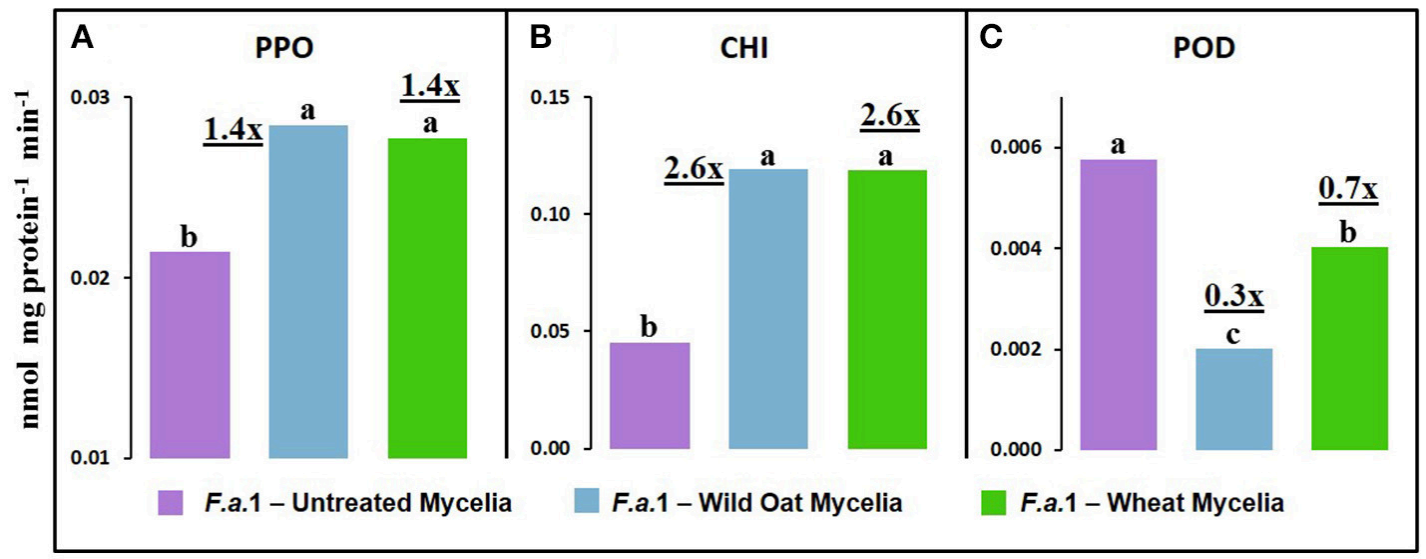
Defense enzyme activities on a protein basis in leachates from *F.a*.1 untreated mycelia, mycelia after exposure to wild oat (*F.a*.1-Wild Oat Mycelia) and mycelia after exposure to wheat (*F.a*.1-Wheat Mycelia). **(A)** polyphenol oxidase, **(B)** exochitinase (CHI), and **(C)** peroxidase (POD). Units are nmol product per mg protein per minute. Bars not having the same letter are significantly different at *P* = 0.05 by LSD. Numbers followed by “x” represent the ratio of activities of *F.a*.1-Wild Oat Mycelia or *F.a*.1-Wheat Mycelia activity to *F.a*.1 Untreated Mycelia.

Leachates were also made from mycelia after exposure to wild oat or wheat caryopses (Figure 8). PPO activity was 1.4-fold greater in leachates of mycelia after exposure to both wild oat and wheat caryopses than in pure mycelial leachates. This can be explained by the release of PPO activity from *F.a*.1-treated caryopses. CHI activity was 2.6-fold greater in leachates of mycelia after exposure to both wild oat and wheat caryopses than in pure mycelial leachates so more of this activity should be attributed to mycelial origin. POD activity was actually inhibited in leachates of mycelia after exposure to caryopses relative to pure mycelial leachates; therefore, it would be logical to primarily attribute POD activity primarily to caryopses in earlier discussed assays (Figure 4).

## Discussion

### Seed decay

The *F.a*.1 isolate was selected from among dozens of fungal strains because it was the most active in causing wild oat caryopsis decay (De Luna et al., 2011). Therefore, the susceptibility of wild oat to decay (Figure 1) was expected. The resistance to decay in wheat var. RL4137 (Figure 1) has not been reported previously. *F.a*.1 therefore demonstrates the selective decay of a weed in a crop that may have potential for managing the weed seed bank in the field. Studies are currently underway to determine if this organism causes wild oat decay in soil. Another factor contributing to wheat resistance to decay was germination, starting at day 4 (Figure 1), which is a previously noted mechanism for escaping decay (Dalling et al., 2011). Our results contradicted our hypothesis that higher levels of enzymes would be associated with greater resistance to decay in these two genotypes since defense enzyme activities were greater in the more susceptible wild oat, compared to wheat. It is likely that the induction of PPO, POD, and CHI is part of a general host pathway triggered by biotic stress (Dixon et al., 1994; Gatehouse, 2002). It should be noted that generalizing about differences between wild oat and wheat is not possible because only one genotype of each was tested here. The point of including wheat was to see whether responses were qualitatively similar in two species, and although defense enzyme responses were similar, decay responses were dissimilar in these two species.

### Caryopsis enzyme activities and protein

As previously mentioned, the relative degrees of induction of PPO, CHI, and POD activities were consistently greater in leachates than in whole caryopses of both species (Figures 2–4). These results confirm our previous report that a disproportionate amount of PPO activity in wild oat was released into the leachate (Fuerst et al., 2011). Results here similarly demonstrated that a disproportionate quantity of CHI and POD activities were released from the caryopsis surface. The release of these activities from the caryopsis surface is consistent with their hypothesized role in defense, where they may contribute to plant apoplastic and extracellular defenses or to interference with pathogen attack.

POD assays had extreme levels of variability and consequently there were few significant responses to *F.a*.1 (Figure 4). For instance, the POD coefficients of variability for untreated and *F.a*.1-treated wild oat whole caryopsis assays were 136 and 115%, respectively; the corresponding values for PPO were 31 and 15%, respectively, and for CHI were 13 and 30%, respectively. Some of the variability with POD assays may have been due to disturbance of the caryopsis surface: we have observed that wounding of the caryopsis surface dramatically increases POD activity in a highly variable manner (data not shown), and slight wounding may have occurred when hand-threshing the wheat or removing wild oat hulls and when handling caryopses for procedures described here. We have also observed that even a miniscule protrusion of the radicle, the initiation of germination, is accompanied with a great release of POD activity (data not shown); however, germinating caryopses were carefully excluded for this study.

The absolute enzyme activities were generally greater in whole caryopses than in leachates (Figures 1–4). In the case of PPO, one reason that not all of the activity was present in the leachate assays was that some insoluble activity remains associated with the caryopsis surface and is not readily leached, as previously reported in wheat and wild oat (Fuerst et al., 2006, 2011). It is also possible that more activity would have been solubilized by a longer leaching period; caryopses were leached for 20 min here, vs. 2 h previously for PPO assays (Fuerst et al., 2011). Similarly, CHI and POD enzymatic activities were generally greater in whole caryopses than in leachates (Figures 3, 4), implying that, like PPO, some insoluble activity may be associated with the caryopsis surface. However, in the case of POD activity in *F.a*.1-treated wild oat, leachate activity was greater than whole caryopsis activity (Figure 4A). There are two possible explanations for the latter observation: (1) it is possible that abrasion of the caryopsis surface solubilized some POD during the tumbling action of the 20-min leaching protocol, which would have increased leachate activity relative to whole caryopsis activity; and (2) the great variability with POD assays, discussed above, may have contributed to the relative scale of these assays (Figure 4A).

Protein appeared to be solubilized during *F.a*.1 challenge in leachates (Figure 5). Part of this increased protein content of the leachates included defense enzymes, such as PPO, CHI, and POD, in caryopsis leachate assays (Figures 2–4). The lower protein content of the wheat leachate (Figure 6) was correlated with generally lower enzyme activities for this wheat variety relative to this wild oat isoline (Figures 2–5). We previously reported that apparent cleavage products of PPO had very high enzymatic activity, and we hypothesized that a protease, possibly of fungal origin, was responsible for the release and activation of PPO (Fuerst et al., 2014). Such a protease-mediated release and activation mechanism might apply to other enzymes as well and might explain the increased protein levels observed with *F.a*.1 exposure.

### Defense enzyme activities in mycelial vs. caryopsis leachates

We previously demonstrated the plant origin of induced PPO activity in *F.a*.1-treated wild oat leachate, where proteins with PPO activity were antigenic to a wheat PPO antibody; furthermore, peptide sequences from the same protein bands were identified as PPO by LC-MS (Anderson et al., 2010). Here, we observed that PPO activity on a protein basis was greater in caryopsis leachates than in leachates of untreated *F.a*.1 mycelia (Figure 7A), consistent with this activity originating primarily from caryopses. Also, in leachates of mycelia, after exposure to both wild oat and wheat caryopses, PPO activity was 1.4-fold greater than in pure mycelial leachates (Figure 8A); this small increase in activity can be attributed to a caryopsis source, and therefore PPO activity in other assays primarily came from caryopses (Figure 2). CHI activity was comparable in mycelial and caryopsis leachates (Figure 7B), and this suggests that some portion of the CHI activity measured in caryopsis assays came from mycelia. However, the contribution of caryopses to CHI activity (Figure 3) should not be minimized because of constitutive caryopsis CHI activity and the relatively small quantity of mycelia retained on caryopses. CHI activity was 2.6-fold greater in leachates of mycelia, after exposure to both wild oat and wheat caryopses, than in pure mycelial leachates (Figure 8B) and it seems likely that fungal CHI contributed to this since the level of CHI induction by *F.a*. 1 in caryopsis leachates was much lower (1.3- to 1.4-fold; Figure 7B). The presence of CHI activity in mycelial leachates is not surprising considering that cell walls of fungi such as *Fusarium* spp. are primarily comprised of chitin, and the growth and repair of fungal cell walls would require fungal chitinases (Sahai and Manocha, 1993; Bowman and Free, 2006). POD activity on a protein basis was greater in caryopsis leachates than in leachates of untreated *F.a*. 1 mycelia (Figure 7C), consistent with this activity originating primarily from caryopses. Furthermore, POD activity was actually inhibited in leachates of mycelia, after exposure to both wild oat and wheat caryopses, relative to pure mycelial leachates (Figure 8C); therefore, it would be logical to primarily attribute the induction of POD by *F.a*.1 observed in caryopses (Figure 4) to caryopsis origin. One possible explanation for the apparent decrease of mycelial POD in the presence of caryopses may be due to fungal catalase, discussed below, which might reduce apparent POD activity by reducing H_2_O_2_; however, the latter co-substrate was not likely rate limiting for the POD assay. Results comparing leachates of caryopses and mycelia (Figures 7, 8) are consistent with our suggestion that most caryopsis PPO and POD activities (Figures 2, 4) are probably of caryopsis origin and that fungal CHI activity probably contributed to apparent caryopsis CHI activity (Figure 3), although the caryopsis contribution should not be minimized.

### General discussion

There are numerous reports of induction of defense enzymes, including PPO, CHI, POD, and OxO, by fungi in various plant tissues (e.g., Dumas et al., 1995; Chen et al., 2000; Radjacommare et al., 2004). Indeed, CHI, POD, and OxO are among many established “pathogenesis response proteins” (Ferreira et al., 2007). Our previous work demonstrated *F.a*.1 induction of PPO activity in wild oat caryopses, whole seeds, and caryopsis leachates (Anderson et al., 2010; Fuerst et al., 2011, 2014). We are aware of no other reports on regulation of such defense enzymes in dormant seeds. However, there are two related reports on a similar induction of defense proteins or enzymes, including PPO and POD, in developing spikelets and heads of wheat by the head blight pathogen, *F. graminearum* Schwabe (Mohammadi and Kazemi, 2002; Zhou et al., 2005).

Anderson et al. (2010) utilized LC-MS to identify proteins present in *F.a*.1-treated wild oat leachates. Three SDS-PAGE protein bands were confirmed to be PPO fragments, two bands matched CHI sequences and one band matched an OxO sequence. It was based on these results that we hypothesized that *F.a*.1 exposure would induce these three enzyme activities, in addition to POD. We confirm, here, the constitutive presence of PPO, CHI, and POD activities, as well as their induction by *F.a*.1, in caryopses and leachates (Figures 2–4). We confirm the constitutive presence of apparent OxO activity in whole caryopsis assays, but not in *F.a*.1-treated whole caryopses or in leachates (Figure 5).

The inhibition of apparent OxO activity in response to *F.a*.1 in whole caryopses and the virtual absence of apparent OxO activity in the leachate (Figure 5) was unexpected. In whole caryopsis assays, we hypothesize that the negative effect of *F.a*.1 on apparent OxO activity is either due to a fungal catalase or an inhibitor. A proteinaceous inhibitor was proposed to explain protease activation of soluble wheat OxO (Lane, 2000). In contrast, catalase, if present, would eliminate OxO-generated H_2_O_2_ before it was detected by the coupled enzyme assay. Fungal catalase has been reported in other plant interactions (Zhang et al., 2004; Maksimov et al., 2013) where it likely plays a role in detoxifying plant-generated H_2_O_2_. Although the OxO leachate activities were minimal, they were significantly lower, and actually slightly negative, in *F.a*.1-treated caryopses (Figure 5). In *F.a*.1-treated leachates, we hypothesize that the presence of OxO protein (Anderson et al., 2010) and the virtual absence of apparent OxO leachate activity, might be explained by one or more of the following mechanisms: (1) an OxO inhibitor (Lane, 2000), (2) a fungal catalase, or (3) the presence of an OxO precursor protein (presumably inactive), as reported in a wheat caryopsis leachate (Jerkovic et al., 2010, Table S7).

In addition to the caryopsis leaching study mentioned above, Jerkovic et al. (2010) measured soluble PPO, CHI, POD, and OxO activities in extracts of whole grain wheat, endosperm, and the dissected outer caryopsis layer (pericarp). Similar to our whole caryopsis studies (Figures 2–5), Jerkovic et al. (2010) reported constitutive activities of PPO, CHI, POD, and OxO in whole grain extracts. CHI was present in both endosperm and whole grain, indicating CHI presence throughout grain tissues. However, PPO, POD, and OxO were absent in the endosperm, implying their presence only in the extrinsic tissue layers. This was confirmed by the observation of especially concentrated levels of POD and OxO, but not CHI, activities in the pericarp. Like Jerkovic et al. (2010), Lane (2000) showed that the epidermis, but not other tissues, contained soluble OxO activity, whereas other wheat tissues only contained insoluble OxO activity. The reason these studies reported high levels of soluble OxO activity in the pericarp and epidermis (Lane, 2000; Jerkovic et al., 2010), and yet we observed the absence of apparent OxO activity in leachates (Figure 5), may be that these earlier studies *extracted* the tissues, which may have solubilized OxO activity; this would imply that the constitutive soluble OxO activity is embedded within extrinsic tissues such as the pericarp, and was not freely diffusible under the conditions of our leaching procedures. Indeed, Lane (2000) reported that, other than the extractable soluble OxO activity in the epidermis, OxO activity in wheat caryopses and seedlings was insoluble, which is consistent with our observations of insoluble activity associated with caryopses (Figure 5).

## Conclusions

These findings supported our hypothesis that the defense enzymes, PPO, CHI, and POD would be induced by the seed decay pathogen, *F.a*.1, in both wild oat and wheat caryopses. The pattern of enzyme response to *F.a*.1 was qualitatively but not quantitatively similar, between the wild oat and wheat genotypes evaluated. The level of induction was greater in leachates than in whole caryopses, implying release of a disproportionate amount of activity into the environment. However, our findings were contrary to the hypothesis that OxO activity would be likewise induced and released by *F.a*.1 and the interpretation of these results is more complex, as discussed above. It is unlikely that the specific defense enzymes studied here play a key role in the differential susceptibility to decay by *F.a*.1 in these two genotypes since defense enzyme activities were greater in the more susceptible wild oat, compared to wheat. This suggests that we are observing a small part of a general plant response to biotic stress and that the total picture of this complex interaction between dormant caryopsis and fungus remains to be discerned. Further research is needed to determine whether defense responses of dormant seeds play a role in seed longevity in the soil in agricultural and other ecosystems. Results are consistent with the hypotheses that (1) dormant seeds are capable of mounting complex responses to pathogens, (2) a diversity of defense enzymes are involved in responses in multiple plant species, and (3) it is possible to identify fungi capable of selective decay of weed seeds without damaging crop seeds, a concept that may be applicable to weed management in the field. While earlier work on seed defenses demonstrated the presence of passive defenses, this work shows that dormant seeds are also quite responsive and capable of activating and releasing defense enzymes in response to a pathogen.

## Author contributions

EF, MJ, AP, and PO: wrote the manuscript; EF and MJ: developed methods and conducted the experiments; EF, MJ, and AP: analyzed the data; EF and PO: secured the funding.

### Conflict of interest statement

The authors declare that the research was conducted in the absence of any commercial or financial relationships that could be construed as a potential conflict of interest.
